# Prevalence of Depression Among Infertile Women at Center for Fertility and Reproductive Medicine in Ethiopia: A Cross‐Sectional Study

**DOI:** 10.1002/hsr2.70434

**Published:** 2025-02-17

**Authors:** Zerihun Beyene, Abraham Fessehaye Sium, Genet Gebremedhin, Bethel Dereje

**Affiliations:** ^1^ Department of Obstetrics and Gynecology St. Paul's Hospital Millennium Medical College (SPHMMC) Addis Ababa Ethiopia

**Keywords:** CFRM, depression, depression and infertility, Ethiopia, fertility, infertility

## Abstract

**Background:**

Screening for depression and understanding its predictors is essential as it can inform the type and extent of psychological intervention that needs to be provided before or during infertility treatment. This study aimed to determine the prevalence of depression and associated factors among infertile women who received care at a Center for Fertility and Reproductive Medicine (CFRM) in Ethiopia.

**Methods:**

This was a cross‐sectional study conducted at CFRM in St. Paul's Hospital Millennium Medical College (Ethiopia) on women with infertility over a period of 3 months, from November 11, 2019 to February 2020. Data were collected prospectively through face‐to‐face interviews with the study participants using PHQ‐9. Data were analyzed using SPSS version 23. Simple descriptive statistics and bivariate and multivariate regression analysis were performed as appropriate. Frequency, percentage, adjusted odds ratio (AOR) along with 95% CI were used to present the finding.

**Results:**

A total of 381 infertile women were approached, and 32 women did not respond, constituting a repose rate of 91.6%. Among the 349 infertile women included in the final analysis, the prevalence of depression was found to be 42.1% (147/349). Low monthly income (AOR = 4.4, 95% CI, 1.3−14.5), unemployment (AOR = 4.3, 95% CI, 1.2−11.5), history of divorce (AOR = 4.4. 95% CI, 1.4−13.0), and partner's low level of education (AOR = 3.2, 95% CI, 1.2−8.9) were associated with detection of depression among the infertile women included in the study.

**Conclusion:**

The prevalence of depression among infertile women is lower than reports from previous studies. Regardless of this relatively low prevalence, our study supports integration of depression screening and psychological treatment for those affected before and during infertility treatments, as a vital component of healthcare infertile women.

## 1 Introduction

Described as the infertility belt region, some parts of sub‐Saharan Africa, has one of the highest burdens of infertility, 15%−45%, in the world [1, 2, 3, 4]. Compared to data from other sub‐Saharan countries such as Sudan, where the prevalence of infertility was reported recently to be 13% [5], and Cameroon with a 19% prevalence of infertility [6], the burden of infertility in Ethiopia is high according to some recent reports. Secondary analysis data from the Ethiopian Health Demographic Survey (EDHS) shows that the prevalence of infertility in the 12 months before the survey was 24.2% (95% CI, 23.1%–25.3%), majority (90.7%) of which was secondary infertility [7]. Another recent study published in 2022 also shows that the prevalence of infertility in selected public hospitals in Addis Ababa (the Capital city of Ethiopia) was 27.6% (95% CI, 23.2–32.0) [8].

Anxiety and depression has been mentioned as major psychological disorders associated with infertility [9, 10]. Compared to those fertile, those infertile has a higher risk of having depression [10, 11]. From infertility follow‐up to the hopefulness for future to the intensity and continuousness of relationship, depression has a profound effect on the life of infertile individuals [12]. The prevalence of depression among infertile individuals varies in different studies; it ranges from 44% to 68.9% [13, 14, 15].

Understanding predictors of depression is as important as detecting the existence of depression among infertile women, as it can inform the type and extent of psychological intervention that needs to be provided before or during infertility treatment. One large study (*n* = 638) that focused on determining the impact of psychological intervention found that psychological intervention was useful in alleviating depression in infertile couples before they received infertility treatment [16]. Even though a number of studies on the prevalence of infertility and causes of infertility in Ethiopia, very little is known about the psychological impact of infertility among infertile women. Our Hospital (St. Paul's Hospital Millennium Medical College, Ethiopia) being a referral hospital for infertility care at subspecialty care including a public IVF center (Center for Fertility and Reproductive Medicine [CFRM]); most infertility patients visit our hospital after initial frequent work‐ups with some unsuccessful treatment efforts with delayed presentation, which may increase anxiety and depression among infertility patients. For this reason, we hypothesized in our study that the prevalence of depression among infertile women may be high. This study presents the prevalence of depression and associated factors among infertile women who received treatment at a public CFRM in Ethiopia.

## 2 Methods and Materials

### 2.1 Study Design, Study Setting, and Study Period

This study was a cross‐sectional study on the prevalence of depression and associated factors on infertile women who received infertility care at Center for Ferility and Reproductive Medicine (CFRM) in St. Paul's Hospital Millennium Medical College (Ethiopia) over a period of 3 months, from November 11, 2019 to February 11, 2020. St. Paul's Hospital is a leading tertiary hospital in Ethiopia, both in terms of patient care and medical training, with several subspecialty care and training available at the college, among which is reproductive endocrinology and infertility (REI). This hospital owns the only public CFRM in the Horn of Africa with more than 2500 IVFs have been performed at it since its establishment in 2017. The primary objective of this study was to determine the prevalence of depression and associated among infertile women who received infertility care at this public CFRM within St. Paul's Hospital.

### 2.2 Inclusion and Exclusion Criteria

Inclusion criteria were confirmed presence of female infertility, patients willing to participate in the study, and those with and without documented male partner's fertility status. The exclusion criteria were incomplete data and a pre‐existing diagnosis of depression (depression diagnosed before diagnosis of female infertility).

### 2.3 Study Outcomes

The primary outcome of the study was the prevalence of depression. The secondary outcomes were factors associated with the presence of depression.

### 2.4 Data Collection Procedure

Data were collected prospectively via face‐to‐face interviews, using a structured questionnaire prepared in English language that was translated into Amharic and back‐translated back into English. The tool used for the screening of depression among infertile women was the patient health questionnaire (PHQ9) and its validated Amharic version. PHQ‐9 is a nine‐item, 27‐point score, self‐administered, or interviewer‐administered questionnaire and based on the Diagnostic and Statistical Manual of Mental Disorders, 4th Edition (DSM‐IV) criteria. Depression severity was based on the PHQ‐9 scores, which range from 0 to 27. A positive depression screen was defined by one of the following two criteria fulfilled. First, either one or both first two PHQ‐9 questions endorsed as a 2 (more than half the days) or 3 (nearly every day). Second, for questions 1 through 9 you need a total of five or more boxes endorsed within the shaded areas of the form to arrive at the total symptom count (questions 1−8 must be endorsed as 2 or 3; question 9 must be endorsed as 1, 2, or 3). Depression severity was defined as follows: scores of 0−4 defined no or minimal depression, scores of 5−9 defined mild depression, score of 10−14 defined moderate depression, scores of 15−19 defined moderately severe depression, and scores greater than 20 defined severe depression.

Two data collectors were recruited, and a 2 day's training session was provided on the purpose of the study, ethical behaving during data collection and content of the questionnaire. Those patients who screened positive for depression were linked to psychiatry side for better management.

### 2.5 Sample Size Calculation and Sampling Procedure

We calculated the sample size using the sample size calculation for a single population by taking population prevalence (p) of depression based on the previous study was taken as 54% [9], and with a consideration of a margin of error of 0.05 and confidence interval (CI) of 95%. The final calculated sample size was 381.

### 2.6 Data Analysis

Data were analyzed using SPSS version 23. Simple descriptive statistics were used to analyze the sociodemographic and fertility characteristics of the participants. Multivariate regression analysis was performed to determine factors associated with positive screen tests. We performed this to control for multiple covariates and confounders, and not miss important covariates, and by doing that we minimized recall and selection bias. Adjusted odds ratio (AOR) and 95% CI were used to present the results significance.

### 2.7 Ethical Clearance

Ethical approval was obtained from the Institutional Review Board (IRB) at St. Paul's Hospital Millennium Medical College. Written informed consent was obtained from the study participants, and patients' confidentiality was diligently maintained throughout the data collection process.

### 2.8 Transparency Statement

Certain restrictions apply to publicly sharing research data and materials because it contains potentially identifying patient information and it is restricted to maintain patient confidentiality. Any researcher can access the data upon reasonable request from the primary author for purposes of reproducing the results or replicating the procedures.

## 3 Results

In this study, a total of 349 women (225 women with primary infertility and 124 women with a secondary type of infertility) were included in the final analysis after 32 women were excluded based on the inclusion criteria, making a response rate of 91.6% (Table 1). The majority (302/349, 86.6%) of the study participants were in the age range 40−49 years‐old. Most of them (306/349, 87.7%) resided in urban areas while the rest 43 (12.3%) were from rural areas. One‐fifth (73/349, 20.9%) had a history of divorce. More than half (57.3%, 200/349) of the participants had a history of treatment for fertility. Sixty‐four (18.3%) had history of infertility for ≥ 10 years. Only half (176/349, 50.4%) of the study participants had proven male partner's intact fertility. Close to 50% (157/349, 45%) had a monthly income of less than 100 US dollars.

**Table 1:** Sociodemographic and fertility characteristics of infertile women seeking infertility care at national fertility treatment center in Ethiopia, 2019−2020.

Variable name		Frequency	Percent
Age	20−39 years	302	86.6
	40−49 years	47	13.5
Residency	Urban	306	87.7
	Rural	43	12.3
Educational level of the women	Uneducated	40	11.5
	Primary	84	24.1
	Secondary	139	39.8
	College and above	86	24.6
Partner level of education	Uneducated	30	8.6
	Primary	78	22.3
	Secondary	132	37.8
	College and above	109	31.2
Employment status	Unemployed	137	39.3
	Farmer	15	4.3
	Others	30	8.6
	Merchant	75	21.5
	Government employee	92	26.4
Years since marriage	< 2 years	2	0.6
	2−5 years	108	30.9
	> 5 years	239	68.5
Previous history of divorce	Yes	73	20.9
	No	276	79.1
Monthly family income	< 5000 birr ($91.00)	157	45.0
	5000−9999 birr ($91−181)	115	33.0
	≥ 10,000 birr (> $181)	77	22.1
Infertility type	Primary infertility	225	64.5
	Secondary infertility	124	35.5
Duration of infertility	< 5 years	196	56.2
	5−9 years	89	25.5
	≥ 10 years	64	18.3
History of treatment for infertility	Yes	200	57.3
	No	149	42.7
Partner proven fertility	Yes	176	50.4
	No	173	49.6
Number of alive child for secondary infertility (*N* = 124)	No child	46	37.1
	1 child	60	48.4
	2 or more children	18	14.5

Among the study participants, 147 (42.1%) were screened positive for depression (Table 2). From these 147 women, more than half (81/147, 55.1%) of them had mild depression followed by another one‐third (43/147, 29.2%) who had moderate depression. Moderately severe depression and severe depression were assessed in 22 (15%) and 1 (0.7%) participants, respectively. Multivariate regression analysis (Table 3) shows low monthly income (AOR = 4.4, 95% CI, 1.3−14.5), housewife (unemployed) (AOR = 4.3, 95% CI, 1.2−11.5), history of divorce (AOR = 4.4, 95% CI, 1.4−13.0), and partner's low level of education‐primary level (AOR = 3.2, 95% CI, 1.2−8.9).

**Table 2:** Depression screening results among infertile women seeking infertility care at a national fertility treatment center in Ethiopia, 2019−2020.

Variable	Category	Frequency	Percent
Screening for depression	Negative	200	57.9
	Positive	147	42.1
Levels of depression among those who screened positive for depression	Mild depression	81	55.1
	Moderate depression	43	29.2
	Moderately severe depression	22	15.0
	Severe depression	1	0.7

**Table 3:** Multivariable analysis result of factors associated with depression among infertile women seeking infertility care at SPHMMC, Addis Ababa, Ethiopia, 2020.

Variable name		COR (CI)	AOR (CI)
Age	20−29 years	Ref	Ref
	30−39 years	4.8 (2.4−9.4)	3.7 (1.4−9.6)
	40−49 years	8.7 (3.7−20.4)	3.6 (0.9 15.5)
Residency	Urban	Ref	Ref
	Rural	9.0 (3.9−21.0)	1.9 (0.3−12.1)
Educational level of the women	Uneducated	19.4 (7.7−49.3)	0.7 (0.1−4.8)
	Primary	11.6 (5.6−24.0)	1.3 (0.3−5.7)
	Secondary	1.7 (0.9−3.5)	0.5 (0.1−2.1)
	College and above	Ref	Ref
Male partner level of education	Primary	10.7 (5.3−21.4)	3.2 (1.2−8.9)
	Secondary	0.9	0.4 (0.2−1.1)
	College and above	Ref	Ref
Employment status	Housewife (unemployed)	10.0 (5.3−18.9)	4.3 (1.2−115.7)
	Farmer	5.0 (1.6−15.8)	0.5 (0.04−5.4)
	Others	1.5 (0.6−4.0)	0.9 (0.2−4.2)
	Merchant	1.5 (0.7−3.1)	1.2 (0.3−5.2)
	Government employee	Ref	Ref
Previous history of divorce	Yes	19.2 (8.8−41.9)	4.3 (1.4−13.0)
	No	Ref	Ref
Monthly family income	< 5000 birr	18.5 (8.2−41.3)	4.4 (1.3−14.5)
	5000−9999 birr	3.3 (1.4−7.7)	1.7 (0.6−4.8)
	≥ 10,000 birr	Ref	Ref
Infertility type	Primary infertility	2.7 (1.7−4.3)	3.5 (1.5−7.9)
	Secondary infertility	Ref	Ref
Duration of infertility	< 5 years	Ref	Ref
	5−9 years	3.5 (2.1−6.0)	1.8 (0.8−4.1)
	≥ 10 years	33.4 (13.5−82.5)	10.3 (3.2−33.6)
Previous treatment of infertility	Yes	1.6 (1.1−2.5)	0.9 (0.4−1.9)
	No	Ref	Ref
Partner proven fertility	Yes	2.6 (1.7−4.0)	1.1 (0.5−2.4)
	No	Ref	Ref

Abbreviations: AOR = adjusted odds ratio; CI = confidence interval; COR = crude odds ratio; Ref = reference.

## 4 Discussion

Although the prevalence of infertility has been reported as one of the highest within the sub‐Saharan region, there is no previous data on the prevalence of depression among infertile women in the country. We sought to determine the prevalence of depression and associated factors among infertile women attending our subspecialty infertility clinic, Center of Reproductive Medicine in Ethiopia. In the present study, the prevalence of depression among infertile women was lower than reports from previous studies, contrary to our initial hypothesis. Low income, history of divorce, unemployment, and the male partner's low level of education were associated with the occurrence of depression. This data adds new information on the prevalence of depression and its predictors among infertile women, which gives valuable insight into designing appropriate interventions to address the challenges of the psychosocial impact of infertility in the region of sub‐Saharan Africa.

Infertility has a profound psychosocial impact on couples, mainly depression and anxiety, especially in Africa, where high childbearing is given a high value [17]. In this study, the prevalence of depression among infertile women who received infertility treatment was found to be 42.1% (147/349), which is lower than reported in previous studies. For example, a cross‐sectional study of 251 infertile women from Iraq found a depression prevalence of 68.9% among the study participants [13]. A meta‐analysis study (*n* = 2818) from Iran also found an overall 44% (1251/2818) prevalence of depression among infertile couples [14], which is similar to findings of another study conducted in Hungary in which 225 (134 primary infertile and 91 fertile) women were analyzed that found a depression prevalence rate of 44.8% [7]. In the African context, a study from Nigeria in which 110 women with infertility were analyzed found a depression prevalence of 52.7% [17], which is again higher than found in our study. Likewise, a study of 100 infertile women from Ghana reported a 62.0% prevalence of depression with age and duration of infertility being among the predictors of depression [18]. Studies from Kazakhstan report an 80%−90% rate of depression and anxiety among infertility patients who attended public ART clinics, which is higher than found in our study [19, 20].

Although the experience of struggling with infertility is highly distressing for both men and women, women often report greater anxiety and depression. Their distress is further exacerbated by invasive infertility treatments such as IVF, being driven to conceive to avoid social pressure, having poor relationships, and having poor self‐esteem. On the other hand, affectionate relationships with the partner, familial support, intrinsic religiosity, wider life goals, and dispositional optimism are associated with better mental health outcomes, hence buffer infertility‐related depression and anxiety [21]. In the present study, low income (below 100 US dollars per month), history of divorce, long history of infertility (≥ 10 years), unemployment, and male partner's low level of education were associated with the detection of depression. One earlier study found pressure on infertile women by relatives as an independent predictor of depression among Asian infertile women [22].

Among the strengths of this study is appropriate sample size allocation. Non‐comparative study design and missing some important predictors (including pressure from relatives) of depression are the main limitations of our study. Among the explanation for the lower prevalence rate of depression among infertile women in our study could be the fact that the Beck Depression Inventory (BDI) was used in most of the previous studies while the PHQ‐9 applied in our study, which is the most commonly used depression screening tool in the general practice but requires different cut‐off values for different clinical settings and practices using the knowledge transition tool, according to findings of a recent systematic review which analyses the diagnostic accuracy this toll for major depressive disorders. The main reason why we used PHQ‐9 instead of BDI as a screening tool in our study is the fact this tool is the most commonly adopted tool for screening depression among different groups of patients in our settings. This should be noted as one of the limitations of our study. Lack of comparison with a control group (women in the general population) further limits our study's conclusions. Apart from the use of different depression screening tool usage in our study, the fact that majority of Ethiopian population is intrinsic religiosity could explain the low prevalence of infertility in our study compared to findings from other African countries (though we didn't collect data on this). However, this relatively lower prevalence of depression should never underestimate the greater need to address this problem as a whole. In fact, policy makers in Ethiopia should act one stablishing centers for screening, detecting, and managing depression due to infertility, which are easily accessible (preferably in direct connection to infertility clinics). Moreover, community‐based interventions focusing on maintaining affectionate relationships with the partner and proving familial support should be instituted.

## 5 Conclusion

Although the prevalence of depression among infertile women found in our study seems lower than reports of previous studies, the figures are high enough to conclude that psychological treatment following screening for predictors of depression and once depression is detected should be a key component of infertility care for such women. Beyond individual treatment for these women, our findings also indicate that increasing community awareness, including discouraging pressure by relatives on such women, should be acted upon to thwart the undesired effects of depression on the women's health. The relationship between depression and fertility treatment outcomes should be further explored in future studies.

## Author Contributions

**Zerihun Beyene:** conceptualization, methodology, data curation, supervision, project administration, formal analysis, writing–original draft, writing–review and editing. **Abraham Fessehaye Sium:** writing–original draft, writing–review and editing. **Genet Gebremedhin:** writing–original draft, writing–review and editing. **Bethel Dereje:** conceptualization, methodology, validation, data curation.

## Ethics Statement

A formal Ethical clearance letter was obtained from the Institutional review board of our hospital (St. Paul's Hospital Millennium Medical College). Written informed consent was obtained from study participants.

## Conflicts of Interest

The authors declare no conflicts of interest.

## Transparency Statement

The lead author, Zerihun Beyene, affirms that this manuscript is an honest, accurate, and transparent account of the study being reported, that no important aspects of the study have been omitted, and that any discrepancies from the study as planned (and, if relevant, registered) have been explained.

## Data Availability

The data that support the findings of this study are available from the lead author upon reasonable request.
